# The dUTPase Enzyme Is Essential in *Mycobacterium smegmatis*

**DOI:** 10.1371/journal.pone.0037461

**Published:** 2012-05-24

**Authors:** Ildiko Pecsi, Rita Hirmondo, Amanda C. Brown, Anna Lopata, Tanya Parish, Beata G. Vertessy, Judit Tóth

**Affiliations:** 1 Institute of Enzymology, RCNS, Hungarian Academy of Sciences, Budapest, Hungary; 2 Queen Mary University of London, Barts and the London School of Medicine and Dentistry, London, United Kingdom; 3 Department of Applied Biotechnology and Food Sciences, Budapest University of Technology and Economics, Budapest, Hungary; Institut de Pharmacologie et de Biologie Structurale, France

## Abstract

Thymidine biosynthesis is essential in all cells. Inhibitors of the enzymes involved in this pathway (e.g. methotrexate) are thus frequently used as cytostatics. Due to its pivotal role in mycobacterial thymidylate synthesis dUTPase, which hydrolyzes dUTP into the dTTP precursor dUMP, has been suggested as a target for new antitubercular agents. All mycobacterial genomes encode dUTPase with a mycobacteria-specific surface loop absent in the human dUTPase. Using *Mycobacterium smegmatis* as a fast growing model for *Mycobacterium tuberculosis*, we demonstrate that dUTPase knock-out results in lethality that can be reverted by complementation with wild-type dUTPase. Interestingly, a mutant dUTPase gene lacking the genus-specific loop was unable to complement the knock-out phenotype. We also show that deletion of the mycobacteria-specific loop has no major effect on dUTPase enzymatic properties *in vitro* and thus a yet to be identified loop-specific function seems to be essential within the bacterial cell context. In addition, here we demonstrated that *Mycobacterium tuberculosis* dUTPase is fully functional in *Mycobacterium smegmatis* as it rescues the lethal knock-out phenotype. Our results indicate the potential of dUTPase as a target for antitubercular drugs and identify a genus-specific surface loop on the enzyme as a selective target.

## Introduction

Despite over a century of extensive research, tuberculosis (TB), the infectious disease caused by *Mycobacterium tuberculosis (M. tuberculosis)*, still remains a public health problem worldwide. It has been estimated that more than two billion people are latently infected with *M. tuberculosis* and a total of around eight million new cases [1], [2] and 1.6 million deaths occurred in 2010 as reported by the WHO [3]. The emergence of multidrug resistant strains of *M. tuberculosis* [4] as well as the existence of extensively drug resistant TB in more than 40 countries [5] and the global spread of HIV are among the factors underlying the resurgence of TB research [6]. New drugs and second generation vaccines are required to control this deadly human pathogen [7].

**Figure 1:**
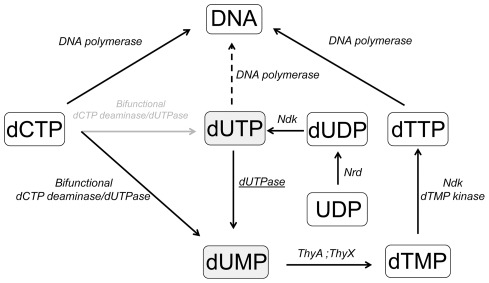
Key enzymes of the *de novo* thymidylate biosynthesis pathway in mycobacteria. Various enzymes present in this pathway are as follows: bifunctional deoxycytidine triphosphate deaminase/ deoxyuridine triphosphate nucleotidohydrolase (bifunctional dCTPdeaminase/ dUTPase), deoxyuridine 5′-triphosphate nucleotidohydrolase (dUTPase), nucleoside diphosphate kinase (Ndk), thymidylate kinase (dTMP kinase), thymidylate synthase (ThyA, ThyX) and ribonucleoside diphosphate reductase (Nrd). The dUTPase enzyme (underlined) converts dUTP (grey highlighted box) into dUMP (grey highlighted box) thereby provides input into dTTP synthesis and eliminates dUTP. An abnormally elevated dUTP/dTTP ratio will lead to uracil incorporation into DNA, as indicated by the dashed arrow. DNA synthesis is provided by several different polymerases (for simplicity no specific polymerases are named here).

An in-depth understanding of the physiological role of enzymes involved in the metabolic pathways of mycobacteria is crucial to identify good targets for rational drug design. Enzymes of the essential thymidylate metabolic pathway are frequently used as targets in anticancer and antimicrobial treatments [8]. The dUTPase enzyme has recently been proposed as a useful target in mycobacteria [9], [10], [11], [12] and in other diseases including cancer and malaria [13], [14], [15], [16]. dUTP is a natural intermediate in the dTTP biosynthetic pathway and is being continuously synthesized in all dividing cells (Figure 1). The enzyme dUTPase is responsible for i) keeping the cellular dUTP/dTTP ratio at a low level to restrict availability of dUTP as a DNA building block and for ii) providing the dTTP precursor dUMP [17], [18], [19], [20].

Decrease in or lack of dUTPase activity may lead to major increase in the uracil content of DNA which resulted in chromosome fragmentation and cell death in the studied cases [21], [22], [23]. Importantly, in all known mycobacterium species, thymidylate biosynthesis necessarily relies on two *de novo* biosynthetic pathways both involving dUTPase action (Figure 1). In addition to the well-known monofunctional dUTPase (Rv2697c), a bifunctional dCTP deaminase/dUTPase (Rv0321) (earlier suggested to exist only in Archea [24], [25]) is also encoded within the *M. tuberculosis* genome [26]. This bifunctional enzyme catalyses both the dCTP deamination reaction and the triphosphate hydrolysis of the resulting dUTP directly producing dUMP from dCTP [27] (Figure 1). Curiously, this bifunctional enzyme has only been reported in *M. tuberculosis* of all mycobacterium species so far. In contrast to mycobacteria, humans encode the dCMP deaminase and the thymidine kinase genes thus providing two alternatives for the dUTPase-mediated [9] pathway.

Previous studies have demonstrated the essentiality of the generally occurring monofunctional dUTPase (product of the *dut* gene) in *E. coli* [28] and in yeast [29], [30]. High density mutagenesis studies suggested that *M. tuberculosis* requires the product of *dut* but not the bifunctional enzyme for growth [31], [32]. In contrast to the thorough biochemical characterization of the *M. tuberculosis* dUTPase enzyme [10], [11], [12], [33], no detailed information has yet been published about the physiological effect of *dut* deletion mutants in mycobacteria. We therefore directed our efforts to obtain formal genetic proof of the essentiality of dUTPase in *Mycobacterium smegmatis* (*M. smegmatis*). We assessed whether the fast growing and non-hazardous *M. smegmatis* can serve as a valid model for *M. tuberculosis* in the investigation of the thymidylate synthesis pathway and chose this organism to carry out the functional deletion of *dut*.

Although dUTPase is often proposed as a drug target [13], [14], a potential problem in selective drug design against this enzyme is the high sequence and structure similarity between the human and pathogen dUTPases. Therefore, we specifically investigated a mycobacteria-specific insert both in enzymatic and phenotypic studies.

## Results

### M. smegmatis and M. tuberculosis share a similar set of enzymes for thymidylate metabolism as revealed by a comparative genomic approach

**Figure 2:**
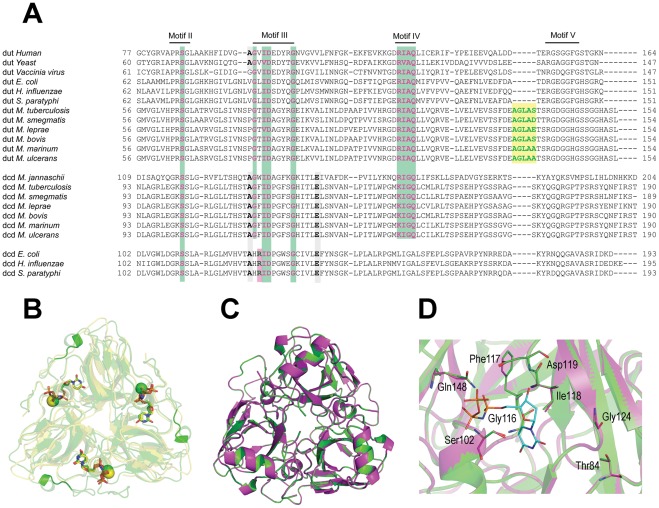
Sequence and structural comparison of selected members of the dUTPase superfamily. (**A**) Conserved motifs are indicated above the sequences as lines. Representative organisms from widely different evolutionary branches are also included for comparison. Mycobacterial dCTP deaminases contain all those conserved residues that are indispensable for dUTPase reaction. Residues conserved between dUTPases and bifunctional dCTP deaminase/dUTPases are important for the dephosphorylation reaction and indicated with green boxes. Residues important for the deamination reaction and crucial for dCTP deaminase monofunctionality are depicted as gray and magenta boxes, respectively. Mycobacterial dUTPases contain an insert present solely in the mycobacterial *dut*, this insert is shown as a yellow box. The alignment was performed with ClustalW. (**B**) The mycobacterial insert induces a loop structure on the surface of the dUTPase monomer. The superimposed structure of hDUT (PDB ID: 3EHW, under publication in a separate paper) and mtDUT (PDB ID: 2PY4) are depicted as yellow and green cartoon representation, respectively. The mycobacterial insert can be seen as cartoon tube representation. In the active sites the bound ligands, dUPNPP and Mg^2+^ can be seen, whereby the Mg^2+^ is visualized as a yellow (hDUT) or green (mtDUT) sphere while dUPNPP is represented as sticks with atomic coloring (carbons in yellow and green). Structures were prepared using PyMol. (**C**) Superimposed overall structure of the *M. tuberculosis* bifunctional dCTP deaminase/dUTPase and the *M. smegmatis* dCTP deaminase enzymes in green and magenta cartoon representation, respectively. (**D**) Enlarged view from **C** showing the conserved residues and the non-hydrolysable substrate analog α-β-imido-dUTP (dUPNPP) as modeled to the active site. Residues and the dUPNPP molecule are in stick representation with atomic coloring (green, magenta and cyan carbons for *M. tuberculosis*, *M. smegmatis* enzymes and dUPNPP, respectively). Note the closely identical organization of both the overall structure and the active site in *M. tuberculosis* and *M. smegmatis* dUTPases.

Comparison of the amino-acid sequences of enzymes involved in thymidylate biosynthesis (shown in Figure 1) revealed that *M. smegmatis* encodes the same enzymes as *M. tuberculosis* does (Table 1), while no homologs of human dCMP deaminase (Uniprot: P32321) or human thymidine kinase (Uniprot: P04183) were identified in these genomes. The role of dCMP deaminase and thymidine kinase in other organisms is to provide the major flux of dUMP production and thymidine salvage, respectively. Such alternative pathways do not seem to exist in mycobacteria, reinforcing the emphasis on dUTPase. In addition, a ClustalW sequence alignment of the C-termini of dUTPases from widely different species exposes a five amino acid long insert that distinguishes mycobacterial dUTPases from the human and other homologs (Figure 2A, B).

**Table 1:** Homology of *M. tuberculosis* and *M. smegmatis* proteins present in the thymidylate synthesis pathway.

Enzyme	Identities^1^ (%)	Similarities^2^ (%)	Gene name in Mtb	Gene name in Msm
Bifunctional dCTP deaminase/dUTPase	87	95	Rv0321	MSMEG_0678
dUTPase	85	94	Rv2697c	MSMEG_2765
Nucleoside diphosphate kinase Ndk	80	88	Rv2445c	MSMEG_4627
Thymidylate (dTMP) kinase	64	71	Rv3247c	MSMEG_1873
Thymidylate synthase ThyA	87	92	Rv2764c	MSMEG_2670
Thymidylate synthase ThyX	86	92	Rv2754c	MSMEG_2683
Ribonucleoside diphosphate reductase NrdE	92	97	Rv3051c	MSMEG_1019, MSMEG_2299
Ribonucleoside diphosphate reductase NrdF2	92	95	Rv3048c	MSMEG_1033, MSMEG_2313

1 =  % identical amino-acids;

2 =  classified on the basis of chemical properties (e.g. polar vs. non-polar) of the respective amino-acids side chains.

Similar Blast search and sequence comparison in the *Mycobacterium leprae*, *Mycobacterium ulcerans*, and *Mycobacterium bovis* pathogens indicated that these species also encode all known enzymes of thymidylate metabolism in mycobacteria sharing above 84% identity. Thymidylate kinase is an exception; its amino acid sequence is more variable between different species (around 66% identity).

In conclusion, we postulate that the thymidine metabolism enzymes of *M. smegmatis* are highly similar not exclusively to *M. tuberculosis*, but also to other mycobacterial pathogens.

### The dCTP deaminase of M. smegmatis is presumably a bifunctional dCTP deaminase/dUTPase

A previous study demonstrated that the enzyme annotated as “dCTP deaminase” in *M. tuberculosis* functions also as a dUTPase [26]. It is most relevant for our study to examine whether *M. smegmatis* and *M. tuberculosis* encodes an identical enzyme set for dUMP production. Therefore, we carried out multiple sequence-alignments for dUTPases, putative and confirmed bifunctional dCTP deaminase/dUTPases and dCTP deaminases from various species. These sequence comparisons show that the mycobacterial enzymes annotated for dCTP deaminases contain conserved amino acid residues that are indispensable for dUTPase activity (Figure 2A). According to Helt *et* *al*. these residues are: Ser102, Asp119 and Gln148 (*M. tuberculosis* numbering, [26]) All of these residues are conserved in mycobacterial dCTP deaminases, dUTPases and the confirmed bifunctional dCTP deaminase/dUTPase enzymes from *M. tuberculosis* and *Methanocaldococcus jannaschii* (Figure 2A). One of the residues indispensable for dUTP hydrolysis is Asp119, which coordinates the catalytic water molecule and interacts with the 3′-OH of the bound nucleotide in dUTPases [34]. As seen in Figure 2A, this residue is conserved in the whole superfamily. The monofunctional dCTP deaminases, however, contain a conserved Arg (Arg126 in *E. coli*) residue (outlined by pink in Figure 2A) which occupies the position of the nucleophile water molecule and forms a salt bridge with the Asp in the catalytic position (Asp128 in *E. coli* [35]). That is why dUTP hydrolysis in monofunctional dCTP deaminases cannot occur. The position of this Arg is occupied by an aromatic residue (Phe, Trp) in dUTP hydrolyzing bifunctional enzymes (Figure 2A), similarly to the putative mycobacterial bifunctional dCTP deaminase/dUTPases.

In the next step, we built the 3D structure of the *M. smegmatis* dCTP deaminase by homology modeling using the *M. tuberculosis* bifunctional dCTP deaminase/dUTPase as template (87% sequence identity). The Ramachandran plot of the homology model shows that nearly 90% of the residues are in the most favored regions and less than 1% of the residues can be found in the disallowed regions which indicate the excellent reliability of the model (data not shown). The overall 3D structures of the *M. tuberculosis* and *M. smegmatis* enzymes are highly similar as seen in Figure 2C which is also supported by the 0.33 Å RMSD value comparing all atoms of the proteins. Importantly, the residues characteristic to bifunctional dCTP deaminase/dUTPase enzymes [26], [35] are in identical positions in *M. smegmatis* and *M. tuberculosis* (Figure 2D). In summary, data presented in Figure 2 indicates that *M. smegmatis* and other mycobacteria likely possess a bifunctional dCTP deaminase/dUTPase enzyme.

### The dut gene is essential for growth in M. smegmatis

**Figure 3:**
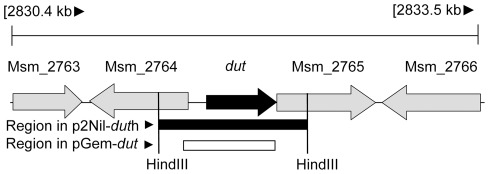
Genomic environment of the *dut* gene. Arrangement of the neighboring genes on the chromosome of the mc^2^ -155 *M. smegmatis* strain is shown together with the regions amplified for the construction of p2Nbk-*dut*h and pGem-*dut*. Relevant restriction sites are also shown. The chromosomal location of the *dut* gene is represented by black arrow, the region cloned into the delivery vector (p2Nbk-*dut*h) is indicated with a black rectangle, and the region cloned in the complementing vector (pGem-*dut*) is shown by a white rectangle.

**Figure 4:**
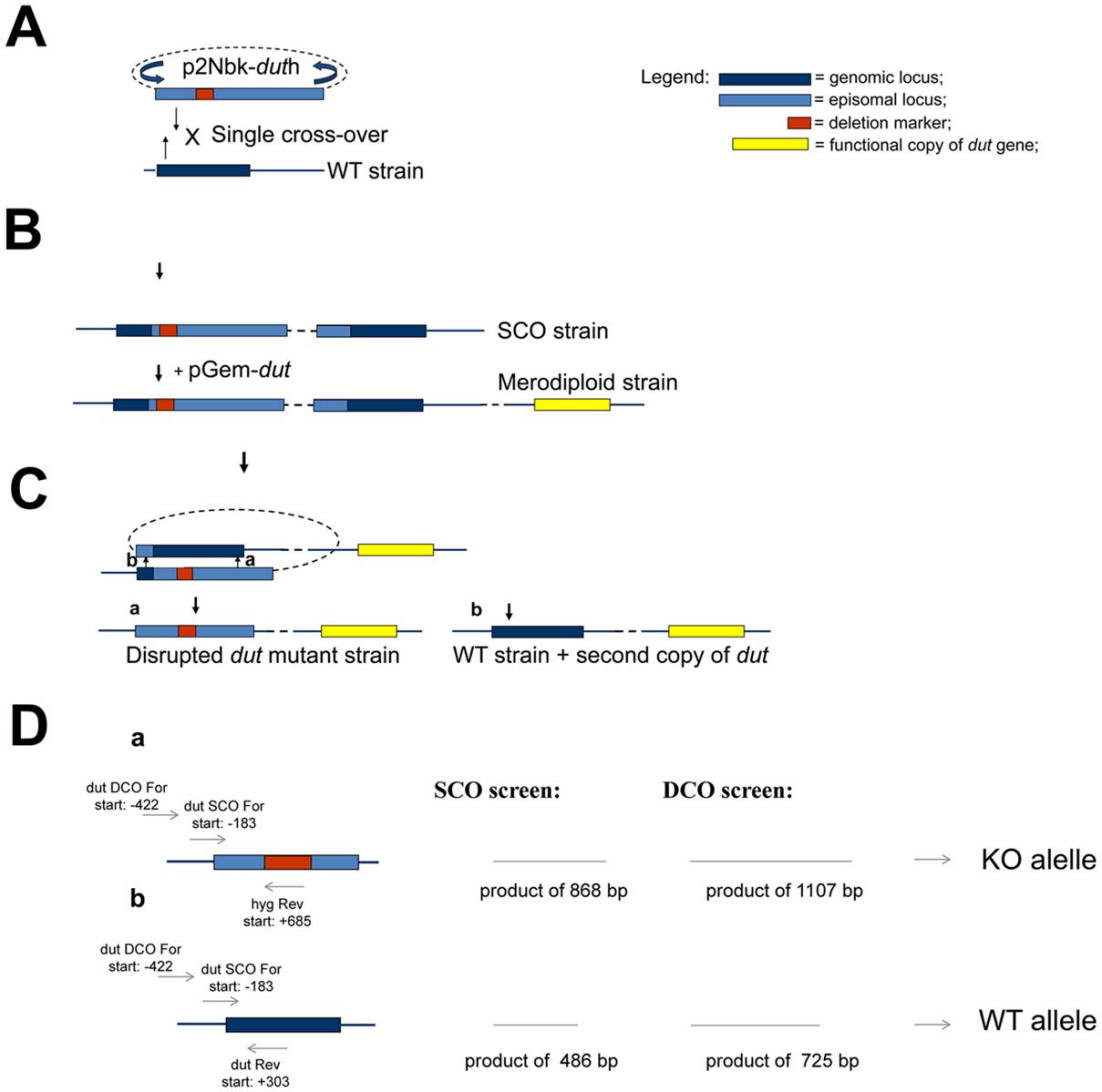
Schematic representation of allelic replacement by homologous recombination . (**A**) Generation of SCO strains. p2Nbk-*dut*h was electroporated into WT competent *M. smegmatis*, and single-crossover (SCO) transformants were selected. (**B**) Merodiploid strains were constructed by electroporating the complementing plasmid (pGem-*dut*) into the SCO strains. (**C**) Generation of disrupted *dut* deletion mutant strain. The double crossover event may result either a disrupted *dut* deletion mutant strain (a), or a wild type strain (b). (**D**) Strategy for SCO and DCO screening. a) shows primers and expected PCR products for the knock-out (KO) allele while b) shows the same for the WT allele. Abbreviations: WT; wild type; SCO; single crossover; DCO; double cross over.

**Figure 5:**
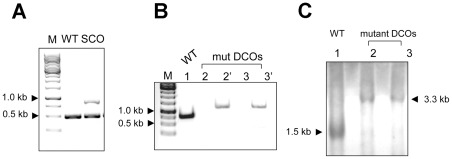
The *dut* gene is essential in *M. smegmatis*. M stands for the 1 kb DNA marker from Fermentas. (**A**) Identification of SCO strain by colony PCR. SCO strains were generated by homolog recombination of p2Nbk-*dut*h with chromosomal copy of *dut*. Chromosomal DNA from *M. smegmatis* mc ^2^-155 was used as a positive control yielding the 486 bp fragment (lane WT); the suicide vector integration due to single-crossover event yielded the 860 bp fragment. (lane SCO). (**B**) Colony PCR analysis of the generated double crossover (DCO) strains. For demonstration, only a subset of 19 samples are shown here. The identical numbers represent samples from the same cell line. The potential DCO cell lines were screened for both the WT copy (indicated as 2, 3) and for the disrupted deletion mutant *dut* gene (labeled as 2′ 3′). The lengths of the expected PCR product for the wild type (WT) *dut* gene and for the disrupted *dut* mutant were 0.7 and 1.1 kb, respectively. (**C**) Southern blot analysis of DCOs. The probe used to perform the hybridization corresponds to the 1.5 kb WT (lane 1) and the 3.3 kb disrupted *dut* deletion mutant (lane 2 and 3) restriction fragment, respectively.

Previous high density mutagenesis study provided the prediction that *M. tuberculosis* requires dUTPase for viability [31] and this finding was further corroborated in a recent genome wide study [32]. In order to address this question directly we used an efficient, reliable two-step deletion strategy [36] to knock out the functional *dut* gene from the chromosome of *M. smegmatis*. Figure 3 shows the chromosomal location and environment of *dut* and the regions used to construct the vectors applied in this study. A schematic representation of the workflow is shown in Figure 4, while the results of the experiments are displayed in Figure 5. We first attempted to construct a marked disrupted deletion mutant of the *dut* gene in the wild-type (WT) background (Figure 4A). A non-replicating delivery vector termed p2Nbk-*dut*h (Figure S1A) was introduced into *M. smegmatis* and single crossover recombinants (SCOs) were selected (see Figure 4B for work-flow and Figure 5A for results). In the next step, double crossovers (DCOs) were generated from the SCOs (Figure 4C) and screened by PCR to determine if the WT or the disrupted *dut* deletion mutant allele was present (Figure 4D). Of the 59 potential DCOs screened 49 were found to be WT DCOs while the other 10 proved to be spontaneous sucrose-resistant (suc^R^) SCO strains (sucrose was used for negative selection). The fact that we could not isolate disrupted deletion mutants in the WT background indicated that the deletion mutant phenotype is probably lethal.

The dUTPase gene, *dut*, is in a predicted operon (http://operondb.cbcb.umd.edu/cgi-bin/operondb/homol_pairs.cgi?gene1_id=219933082&gene2_id=219933083) with an unannotated protein downstream of *dut* (*M. SMEGMATIS_2766*) (Figure 3). Therefore, the inability to isolate deletion mutants in the WT background may have been due to the disruption of the downstream gene of unknown function. To unequivocally confirm that *dut* alone is essential we constructed a merodiploid strain carrying an additional copy of *dut* expressed from its native promoter using a mycobacteriophage L5-based integrating vector (Figure S1B). After confirming the integration of the complementing vector into the SCO strains, DCOs were generated in this background and screened by PCR (Figures 4, 5). Of the 19 potential DCOs screened, 13 were WT while 6 contained the *dut* disrupted allele (Figure 5B). Consequently, *dut* can be disrupted at its native locus if a functional copy of the same gene is supplied elsewhere. The expected genotype of the disrupted deletion mutant strains was confirmed by Southern blot analysis (Figure 5C). These results prove the essentiality of *dut* in *M. smegmatis* as we could only obtain deletion mutants at the native *dut* locus in a merodiploid strain (*p*<0.00025, using Fisher's exact test).

### M. tuberculosis and M. smegmatis dUTPases are functionally equivalent

**Figure 6:**
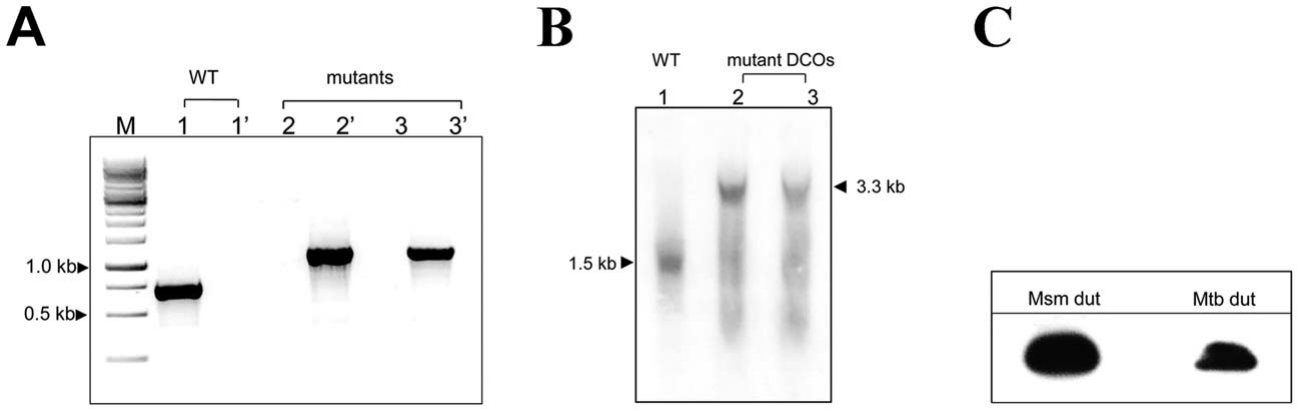
The *M. tuberculosis* dUTPase is able to complement the lethal phenotype in *M. smegmatis* . (**A**) Colony PCR analysis of the generated DCO strains. For demonstration, only a subset of 20 samples are shown here. M stands for the 1 kb DNA marker from Fermentas. The identical numbers represent samples from the same cell line. Every cell line was screened for both the WT copy (indicated as 1, 2, 3) and for the disrupted deletion mutant *dut* gene (labeled as 1′ 2′ 3′). The lengths of the expected PCR product for the wild type (WT) *dut* gene and for the disrupted *dut* mutant were 0.7 and 1.1 kb, respectively. (**B**) Southern-blot analysis of *dut* disrupted, *M. tuberculosis dut* coding mutants. WT was used for control. The probe used to perform the hybridization corresponds to the 1.5 kb WT (lane 1) and the 3.3 kb *dut* disrupted mutant (lane 2 and 3) restriction fragment, respectively. (**C**) Western-blot analysis of FLAG-tagged *M. tuberculosis* dUTPase expression in *M. smegmatis.*

We previously showed that thymidylate metabolism enzymes share high sequence similarity within the *Mycobacterium* genus (Table 1). To demonstrate functional similarity experimentally, we tried to rescue the lethal phenotype of *dut* disruption with the expression of *M. tuberculosis* dUTPase in the mutant DCO strain. A complement vector carrying the *M. tuberculosis dut* gene was constructed and electroporated into SCO cells. After confirmation of the plasmid integration event, the resulting strains were screened for DCO events. Out of 20 potential DCOs screened, 10 genomic *dut* disrupted mutant cell lines carrying the *M. tuberculosis* protein could be isolated (Figure 6A). We also obtained 9 WT and 1 sucrose-resistant SCO strains. The expected genotype of the disrupted deletion mutant strains were confirmed by Southern blot analysis (Figure 6B). To check if normal protein expression is driven from the complementing plasmids in *M. smegmatis*, we carried out a Western-blot analysis using the FLAG-tag epitopes engineered on the proteins. Our results show that the expression of the dUTPase protein from *M. tuberculosis* and *M. smegmatis* are comparable (Figure 6C). The fact that the *M. tuberculosis dut* could revert lethality in our *M. smegmatis* system suggests that *M. tuberculosis* and *M. smegmatis* dUTPases are functionally equivalent.

### The mycobacteria-specific surface loop is essential for viability

**Figure 7:**
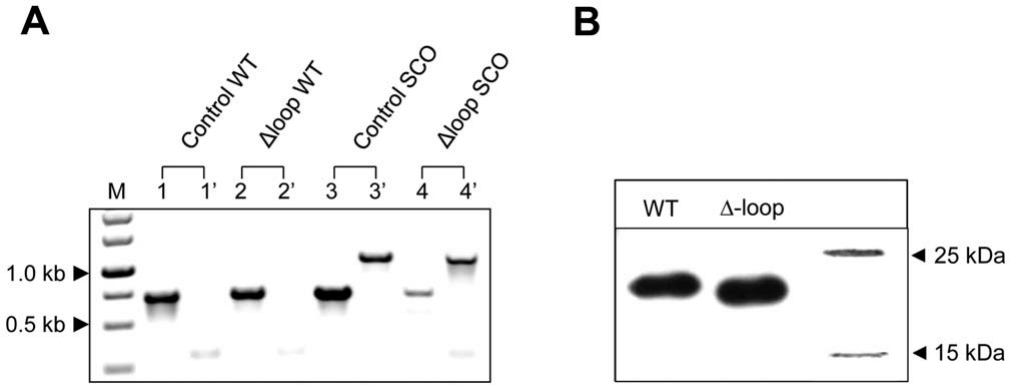
The Δ-loop mutant dUTPase is unable to rescue the lethal phenotype despite its normal expression level. (**A**) Colony PCR analysis of the generated double crossover (DCO) strains. 88 strains were screened and no mutant cell line could be isolated. For demonstration, only a subset of the samples are shown here. M stands for the 1 kb DNA marker from Fermentas. The identical numbers represent samples from the same cell line. Every cell line was screened for both the WT copy (indicated as 1, 2, 3, 4) and for the disrupted mutant *dut* gene (labeled as 1′ 2′ 3′ 4′). The lengths of the expected PCR product for the wild type (WT) *dut* gene and for the disrupted *dut* mutant were 0.7 and 1.1 kb, respectively. (**B**) Western-blot analysis of FLAG-tagged WT and Δ-loop dUTPase expression in *M. smegmatis* transformed with the appropriate construct.

The mycobacteria-specific C-terminal insert in dUTPase presents itself as a unique opportunity to selectively target the mycobacterial protein in a human background. Nevertheless, the functional and the physiological role of this five-aminoacid-insert, although well conserved in mycobacteria, is still unknown. In order to address this intriguing question, we attempted to complement the lethal deletion mutant phenotype with the *dut* gene lacking the mycobacteria-specific insert. For this, a complement vector carrying the *dut* mutation termed Δ-loop was constructed and electroporated into SCO cells. Following the confirmation of the plasmid integration event, the resulting strains were subjected to an extensive DCO screen (Figure 7A). Out of 88 potential DCOs screened no deletion mutant cell line could be isolated. We also compared the protein expression levels in merodiploid strains carrying the WT or the Δ-loop mutant dUTPase complementing copies besides the intact endogenous *dut* gene. The Western-blot on FLAG-tagged dUTPase constructs showed that the expression efficiencies of WT and Δ-loop dUTPases are indistinguishable (Figure 7B). The fact that we could not isolate any viable *dut* deletion mutant in a Δ-loop background strongly suggests that the mycobacteria-specific segment is essential for the growth of *M. smegmatis.*

### The lack of the mycobacteria-specific surface loop results in minor changes in the enzymatic properties of M. tuberculosis dUTPase

**Figure 8:**
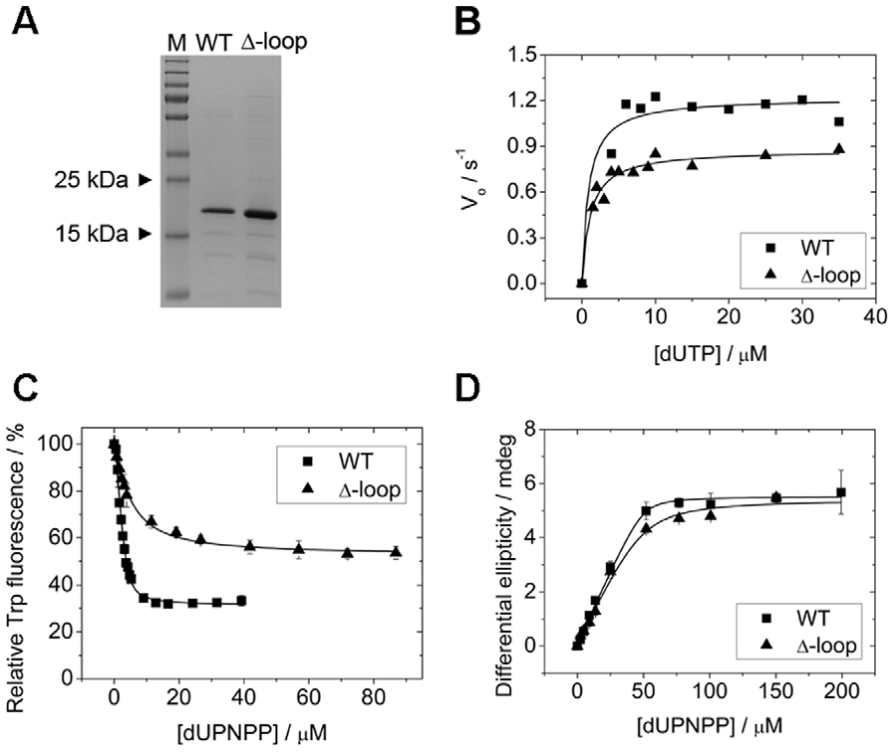
Effect of the Δ-loop mutation on the substrate hydrolysis and binding of *M. tuberculosis* dUTPase. (**A**) SDS-PAGE analysis of the purified proteins used in this study. M stands for the PageRuler Plus Prestained Protein Ladder (Fermentas). The WT and Δ-loop mutant dUTPases have calculated molecular weights of 18.0 kDa and 17.6 kDa, respectively. (**B**) The steady-state activity of WT and Δ-loop mutant dUTPase is shown. Michaelis-Menten curves for the WT (squares) and the Δ-loop mutant (triangle) were measured using the phenol red pH indicator assay. Fitting the Michaelis-Menten equation to the curves yielded the following V_max_ and K_M_ values: 1.22±0.06 s^−1^ and 0.9±0.5 μM for WT, 0.88±0.02 s^−1^ and 1.1±0.2 μM for Δ-loop. (**C**) Fluorescence intensity titration of the WT and the Δ-loop mutant using the single Trp signal is shown upon dUPNPP binding. Smooth lines through the data are quadratic fits yielding the K_d_ values listed in Table 2. Errors represent S.D. for n = 3. For more parameters see Table 2. (**D**) CD equilibrium titrations. Comparison of ligand (dUPNPP) binding to the WT and to the Δ-loop mutant dUTPase. Smooth lines represent quadratic fits to the data yielding the following K_d_ values: 0.9±0.5 μM for WT and 3.9±2.4 μM for Δ-loop.

Upon obtaining the above striking result with the Δ-loop strain we were interested to reveal the enzymatic behavior of the Δ-loop enzyme (mtDUT^Δ-loop^) *in vitro*. Because the Δ-loop strain was not viable, one might presume that the enzymatic activity of mtDUT^Δ-loop^ would be compromised. To investigate the enzymatic efficiency of the mtDUT^Δ-loop^, we expressed and purified the mutant protein (Figure 8A) which proved to be as stable as the WT *in vitro*.

**Table 2:** Kinetic parameters of WT and Δ-loop deletion mutant *M. tuberculosis* dUTPase enzymes and dissociation constants of dUTPase-dUPNPP complexes.

		WT	Δ−loop
**Activity measurement**	k_cat_ (s^−1^)	1.22±0.06	0.88±0.02
	K_M_ (µ M)	0.9±0.5	1.1±0.2
**Fluorescence intensity titrations**	A_max_ (%)	−72±2	−46±1
	K_d_ (µ M)	0.3±0.1	3.3±0.5
**Circular dichroism measurement**	K_d_ (µ M)	0.9±0.5	3.9±2.4

To evaluate the consequences of the loss of the specific insert to the enzymatic cycle, we measured the activity of mtDUT^Δ-loop^ and compared it to that of mtDUT^H145W^ [11] used as WT. As shown in Figure 8B, the maximal steady-state activity of the mtDUT^Δ-loop^ deletion mutant decreased to 0.8 s^−1^ compared to the WT (1.2 s^−1^) while the Michaelis constant (K_M_) was found to fall between 0.9–1.1 µM for both enzymes (Table 2). The observed 1.2-fold decrease in V_max_ and the similar K_M_ values indicate that the catalytic efficiency of dUTPase is little affected by this mutation. The surface loop is relatively close to the active site of the enzyme and it was therefore possible that the lack of it disturbs substrate binding. We investigated this possibility by determining the dissociation constant (K_d_) of the WT and deletion mutant dUTPase complexed with the non-hydrolysable substrate analog α-β-imido-dUTP (dUPNPP) using fluorescence and circular dichroism (CD) titration. We took advantage of the discriminative power of a Trp sensor built in the active site [10], [37] to measure the binding by fluorescence titration. This Trp residue substitutes a conserved histidine residue which overlaps with the uracil ring of the substrate dUTP forming a Π – Π aromatic stacking interaction. It was shown that introduction of a Trp residue to the aromatic site results in WT enzymatic behavior [37]. According to Toth *et al*., the fluorescence signal of the Trp residue changes upon substrate binding which allows for the measurement of the dissociation constant of the enzyme-substrate complex [37]. Figure 8C shows fluorescence intensity titrations upon dUPNPP binding to dUTPase. The mtDUT^Δ-loop^ mutant displays a reduction in the observed fluorescence quench upon ligand binding compared to the WT and yielded about ten times higher K_d_ (Table 2). Titration of the differential CD signal of the enzyme-substrate complex upon dUPNPP binding to mtDUT^H145W^ and mtDUT^Δ-loop^ yielded dissociation constants of 0.9 and 3.9 µM for the WT and for mtDUT^Δ-loop^, respectively (Figure 8D and Table 2). The CD measurements are in line with the fluorescence-based ones in that the mutation slightly affects the substrate binding affinity of dUTPase (4.3–11 fold decrease). It must be noted that the binding affinity of dUPNPP and the cognate substrate dUTP to the enzyme may be different from each other to a small extent. Nevertheless, relative changes tend to be the same regardless of the substrateanalog.

In summary, deletion of the mycobacterium-specific insert had no major effects on dUTPase enzymatic properties *in vitro* despite its essentiality in the living cell.

## Discussion

In the present paper, we investigated the physiological effect of dUTPase gene disruption in *M. smegmatis*. Lethality of dUTPase deletion mutants has been reported before only in *E. coli* [28] and yeast [29], [30]. Current understanding of the mycobacterial DNA repair system is still poor compared with that of other bacterial (e.g. *E. coli*) organisms [38], [39]. It is known, however, that the *M. tuberculosis* and *M. smegmatis* genomes lack several of the DNA repair genes and that *M. tuberculosis* polymerases are highly error-prone [40]. We had thus considered that in case of mycobacteria the importance of preventive DNA repair measures, as e.g. exerted by dUTPase is remarkably high. The results presented here corroborate this assumption. In order to demonstrate the importance of the enzyme dUTPase in mycobacterial viability, we applied a two-step homologous recombination based strategy. We could isolate disrupted deletion mutants solely when a second, functional copy of *dut* was provided, which proves that this enzyme is essential in *M. smegmatis*.

The sequence analysis and homology modeling experiments demonstrated that thymidylate biosynthesis is homologous in all known mycobacteria and therefore, our findings probably apply to all of them. We showed that the mycobacterial dCTP deaminases are well conserved and most likely exert the dCTP deaminase/dUTPase function so far only studied in *M. tuberculosis*. High density mutagenesis reports [31], [32] suggested that the bifunctional dCTP deaminase/dUTPase enzyme is dispensable for viability. Our current study demonstrates that the loss of monofunctional dUTPase is lethal to *M. smegmatis*, nevertheless, lethality may not be due to the lack of dUTPase activity. It is yet to be systematically investigated how dUTP hydrolysis activity exerted by the mono- and bifunctional dUTPases affects viability in mycobacteria.

Comparison of the available dUTPase sequences unambiguously led to the conclusion that mycobacterial dUTPase confer a unique and strictly mycobacteria-specific insert close to the C-terminus of the polypeptide chain (Figure 2A). This insert induces the formation of a surface loop close to the entrance of the active site as can be seen in the crystal structure of *M. tuberculosis* dUTPase (Figure 2B) [10], [12], [41]. We demonstrated that the mycobacteria-specific loop is essential for viability. As this segment of the protein contributes to the physiological effect conveyed by the whole enzyme, it could serve as a powerful selective target surface on the molecule.

Our spectroscopic and steady-state kinetics measurements on the purified mtDUT^Δ-loop^ protein led to the conclusion that the five-aminoacid-insert near the active site does not have a major impact on catalysis itself (Figure 8B, C, D). The enzymatic activity reduced to approximately 67% in mtDUT^Δ-loop^ is not significant compared to the orders of magnitude decrease in activity and increase in K_M_ caused by almost any other mutation we previously introduced to homologous dUTPases [33], [37], [42]. The exact correlation of dUTPase activity with mycobacterial viability is not known. However, Guillet *et al*. reported that mutant yeast strains are viable with dUTPase enzymatic activity reduced to less than 10% of the wild-type one [30]. This finding suggests that mtDUT^Δ-loop^ could probably complement the lethal phenotype if the five-aminoacid-long insert would not be required for other processes.

There are several studies in the literature mapping essential domains to determine protein function in mycobacteria (e.g. the WhiB-like proteins [43] or the UvrD1protein [44]). The majority of the residues investigated in these papers were strictly required for enzyme activity. This is not the case with the mycobacterium-specific dUTPase loop. We speculate that due to its exposed position, it might provide a binding surface for a yet unknown protein partner or another ligand and this interaction might mediate the essential function detected in our assay. A recently published study demonstrated that some bacteriophage dUTPases have two different and genetically distinct activities [45]. It seems that mycobacterial dUTPase may also have functions beyond their enzymatic activity provided by a unique and essential sequence motif. To elucidate the specific function of this short sequence extensive further studies are required.

Key enzymes of the *de novo* thymidine biosynthesis pathway (Figure 1) are attractive targets in the search for novel antitubercular therapeutics. The currently used target enzymes are the essential thymidylate synthase ThyX in *M. tuberculosis* [46], [47], [48] and ThyA in general [8], [49]. Here we provide formal genetic proof of dUTPase essentiality in a mycobacterium for the first time and propose dUTPase as a potential drug target. Based on high sequence similarities and on the functional equivalence of the *M. tuberculosis* and *M. smegmatis* dUTPases, we suggest that the dUTPase enzyme and its mycobacteria-specific loop might bear similar key physiological roles in other, pathogenic mycobacterial species. The pathogens *Mycobacterium leprae*, and *Mycobacterium ulcerans*, the causative agents of leprosy and Buruli ulcers, respectively, still remain a serious problem. Incidences of Buruli ulcers are increasing in certain areas of the tropics such as West Africa [50]. In addition, *Mycobacterium bovis* has a broad host range, producing tuberculosis in several mammals including humans and cattle, having a considerable economic and public health importance in its own right [51].

In summary, we showed that disruption of the *dut* gene results in lethality in *M. smegmatis* and that the mycobacteria-specific insert is required for effective complementation of the lethal phenotype. We also showed that the mycobacteria-specific insert has only subtle contribution to the enzymatic activity of dUTPase and is therefore presumed to mediate an important non-enzymatic function within the bacterium. Finally, an inventory of the mycobacterial enzymes of thymidylate synthesis indicates that mycobacteria share a common dTTP biosynthetic route and that our findings in *M. smegmatis* may be applied to other, pathogenic mycobacterial species.

## Materials and Methods

### Protein sequence analysis and homology modeling

To determine the degree of identity between the enzymes involved in the thymidylate metabolism of *M. tuberculosis* and *M. smegmatis*, we carried out amino acid sequence search and comparisons using the http://blast.ncbi.nlm.nih.gov/Blast.cgi web server and the protein-protein BLAST algorithm. Default parameter settings were applied. Multiple sequence alignments were performed using the ClustalW software. The prediction of the 3D structure of the dCTP deaminase of *M. smegmatis* (Uniprot: A0QQ98) was performed by comparative homology modeling using the SWISS-MODEL Server and Workspace http://swissmodel.expasy.org/ [52]. For template, the apo crystal structure of the *M. tuberculosis* bifunctional dCTP deaminase: dUTPase (PDB: 2QLP) was used (87% sequence identity with the *M. smegmatis* enzyme) [26]. The quality of the generated model was evaluated by the ANOLEA [53], QMEAN [54] and PROCHECK [55] programs.

### Bacterial strains, media and growth conditions

*M. smegmatis* mc^2^155 [56] was grown in Lemco medium (broth) or with the addition of 15 g L^−1^ Bacto agar (solid) as described previously [57]. Kanamycin was added at g/ml, hygromycin B at 100 μg/ml, gentamicin at 10 μg/ml, and streptomycin at 20 μg/ml concentration. For sucrose selection, 5% (wt/v) sucrose was included. X-Gal (5-bromo-4-chloro-3-indolyl-β- D- galactopyranoside) was used at 40 μg/ml.

### Construction of the suicide delivery vector

The suicide delivery vector was constructed to generate the *dut* deletion mutant using a rapid cloning system [36]. First, a 2.1 kb fragment containing the *dut* gene together with its flanking region was amplified by polymerase chain reaction (PCR) from the *M. smegmatis* genome (region indicated in Figure 3). The 2.1 kb fragment was subsequently cloned into p2NIL [36] using the *Hind*III restriction site. The hygromycin marker gene (*hyg*) was PCR amplified from the pGOAL19 plasmid [36] using primers that carry the AgeI restriction sites. The marked disrupted allele was subsequently constructed by inserting the 1.8 kb *hyg* gene into the single AgeI site in the middle of the *dut* gene, resulting in a disrupted, non-functional dUTPase. The 6.1 kb PacI cassette carrying the *lacZ* and *sacB* selection markers from pGOAL17 [36] was cloned into the sole PacI site of p2NIL to yield p2Nbk-*dut*h. Primers used for cloning, mutagenesis and screening are compiled in Table S1.

### Construction of the complementing vectors

To make the complementing construct for the *M. smegmatis* WT *dut*, PCR was used to amplify the complete gene together with the native promoter 337 bp upstream of dut. Thereafter the 0.8 kb PCR product was A-tailed and cloned into pGEM T-Easy (Promega). The Gm-Int *Hind*III cassette from the pUC-Gm-Int plasmid [58] was introduced into the resulting construct to yield the integrating vector pGem-*dut*. The Δ-loop deletion mutant *dut* complementing vector was made by the QuikChange method (Stratagene) using the pGem-*dut* as template. The *M. tuberculosis dut* coding complementing vector was made by exchanging the *M. smegmatis dut* coding sequence for the *M. tuberculosis dut* coding sequence. A FLAG-tag was cloned into all vectors subsequently. All sequences were verified by restriction digestion and sequencing.

### Generation of SCO, DCO and merodiploid strains

5 μg of UV pretreated plasmid DNA [59] was electroporated into competent *M. smegmatis* [60], then single-crossover (SCO) transformants were selected on medium containing kanamycin, hygromycin and X-Gal. Merodiploid strains were constructed by electroporating the SCO strains with the appropriate complementing plasmids followed by isolation of kanamycin-, hygromycin-, and gentamicin-resistant transformants. Double crossovers (DCOs) were generated in the wild-type and merodiploid background by streaking cells onto plates lacking antibiotics. DCO selection was performed on medium containing sucrose, X-Gal, and gentamicin as required [36]. Colony PCR screening was carried out using gene-specific screening primers (Table S1) and Red-Taq polymerase (Sigma Aldrich) to determine whether the wild-type or the deletion mutant allele was present in the targeted chromosomal location.

### *Genomic DNA isolation* was carried out as follows

10 mL liquid culture of *M. smegmatis* was harvested, and the cells were resuspended in 1 mL 10 mM Tris pH 7.5. Thereafter 0.1 mm glass beads were added to 2 mL volume, the cells were disrupted by vortex and ice incubation by turn. After centrifugation the supernatant was manipulated routinely to purify DNA by phenol:chloroform:IAA (25∶24∶1) extraction followed by isopropanol precipitation [61].

### *Southern blot analysis* was carried out using the DecaLabel^TM^ DNA Labeling Kit (Fermentas) according to the manufacturer's instructions

Restriction digestion of the genomic DNA was performed using NcoI and PstI resulting in 1.5 kb and 3.3 kb fragments in the case of WT and *dut*-disrupted mutant strains, respectively. The probe was a 0.7 kb fragment encompassing the *dut* gene (for primers see Table S1).

### Verification of protein expression from the complement vector

1 μg FLAG-tagged pGEM vector carrying the WT or the Δ-loop *M. smegmatis*, or the WT *M. tuberculosis dut* was electroporated into competent *M. smegmatis* cells. Gentamycin-resistant transformants were isolated and the integration of the complementing vector was confirmed by PCR reaction. FLAG-tagged *dut* coding strains were grown until the OD_600_ reached 0.4–0.5 then the cells were harvested by centrifugation. Pellets were resuspended in lysis-buffer (50 mM Tris-HCl, pH = 7.5; 140 mM NaCl; 1 mM EDTA; 0.5% SDS; 1% Triton X-100; 0.5 mM PMSF; 2 mM BA; 15 mM β-mercaptoethanol; 0,1 mg/ml DNase) and sonicated (Elma, S30H ElmaSonic, D78224) for 4 times 5 minutes. Concentrations of the final supernatants of the cell extraction were measured using Nanodrop ND-1000 and equalized by dilution before Western-blot analysis. Protein lysates were heated at 95°C for 5 min, separated by SDS-PAGE, and transferred to PVDF membrane for immunoblotting with the specific antibody against FLAG-tag (Sigma, Monoclonal ANTI-FLAG® M2 antibody). Immune-complexes were visualized using enhanced chemilumincesence. The blotted polyacrylamide gel stained with Comassie Brilliant Blue and the PVDF membrane stained with Ponceau were used as loading controls.

### Mutagenesis, cloning and dut gene expression

Site-directed mutagenesis was carried out according to the Stratagene QuikChange site-directed mutagenesis instructions and verified by sequencing of both strands. The Δ-loop deletion mutant (mtDUT^Δ-loop^) was created by deletion of the five (Ala133-Ser137) loop-specific amino acids (for mutagenic primers see Table S1). The recombinant dUTPase carrying an N-terminal hexa-His tag was cloned into pET19-b vector and expressed in *Escherichia coli* BL21(DE3) (pLysS) cells. For protein overexpression, the cells were grown to an OD_600_ of 0.4, treated with 0.5 mM isopropyl-β-D-thiogalactopyranoside at 37°C for 3 hours.

### Protein purification was carried out as described previously [41]

The final supernatant after cell extraction was loaded on a Ni-NTA column (Novagen) and purified according to the Novagen protocol. The purity of the protein preparation was analyzed by SDS-PAGE. The enzyme conferring a single Trp in the active site (mtDUT^H145W^) was used as wild-type in the kinetic measurements [10]. Protein concentration was measured using the Bradford method (Bio-Rad Protein Assay) and by UV absorbance (λ_280_ = 8480 M^−1^cm^−1^ for mtDUT^H145W^and for mtDUT^Δ-loop^) and is given in monomers. All measurements were carried out in the dialysis buffer comprising 20 mM HEPES pH 7.5, 100 mM NaCl, 2 mM MgCl_2_ and 1 mM DTT if not stated otherwise.

### Steady-state colorimetric dUTPase assay

Protons released in the dUTPase reaction were detected by phenol red pH indicator in 1 mM HEPES pH 7.5 buffer also containing 100 mM KCl, 40 μM phenol red (Merck) and 5 mM MgCl_2_. A Specord 200 (Analytic Jena, Germany) spectrophotometer and 10 mm path length thermostatted cuvettes were used at 20°C. Absorbance was recorded at 559 nm. The Michaelis-Menten equation was fitted to the steady-state curves using Origin 7.5 (OriginLab Corp., Northampton, MA).

### Fluorescence intensity titrations

Fluorescence was measured in a Jobin Yvon Spex Fluoromax-3 spectrofluorometer at 20°C, with excitation at 295 nm (slit 1 nm) and emission at 347 nm (slit 5 nm). 4 μM protein was titrated by the addition of 1–2 μl aliquots from concentrated dUPNPP solutions (purchased from Jena Bioscience, Germany). Because large concentrations of nucleotides were used, care was taken to correct for any additional fluorescence or inner filter effect imposed on the measured intensities by the nucleotide stock solutions.

### Circular dichroism intensity titrations

CD spectra were recorded at 20°C on a JASCO 720 spectropolarimeter using a 10 mm path length cuvette. 50 µM protein was titrated by stepwise addition of the non-hydrolysable substrate analogue dUPNPP, in a buffer containing 20 mM HEPES pH 7.5, 50 mM NaCl and 2 mM MgCl_2_. A spectrum between λ = 240–350 nm was recorded at each nucleotide concentration. Differential curves were obtained by subtracting the signal of dUPNPP alone from that of the corresponding complex. Differential ellipticity at λ_max_ = 269 nm was plotted against the dUPNPP concentration to obtain the binding curves. The following quadratic equation was fitted to the experimental curves:
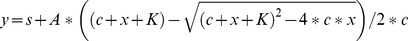
s = y at x = 0; A  =  amplitude; c  =  protein concentration; K = K_d._

### Statistical analysis

Spectroscopy and kinetics measurements were carried out at least 3 times. Error bars represent standard deviations. In case of no error bars shown, a representative curve is displayed and the relevant table shows the standard deviations of a certain parameter obtained from several different measurements. In case of experiments carried out in the whole bacterium the Fischer's Exact Test was applied to determine the *p* value.

## Supporting Information

Figure S1 **Key plasmids used in the generation of** ***dut*** **deletion mutant** ***M. smegmatis*** **.** (**A**) p2Nbk-*dut*h delivery vector used to generate mutant SCOs. The 2.1 kb HindIII *M. smegmatis* fragment indicated in Figure 3 was inserted into the p2NIL vector to construct the delivery vector. The *dut* allele was disrupted with a 1.8 kb fragment encoding hygromycin resistance, resulting in a non-functional *dut* gene. (**B**) The plasmid pGem-*dut* was used to complement the gene-disruption mutation. The wild-type *dut* allele together with its own promoter (337 bp upstream of the *dut* coding region) was cloned into an L5-based integrating vector to produce pGem-*dut*. Detailed cloning procedures are given in Materials and Methods. C*dut*, WT *dut* gene with its own promoter; *kan*, kanamycin resistance gene; *hyg*, hygromycin resistance gene; *lacZ*, β – galactosidase; *sacB*, sucrose sensitivity gene; *amp*, ampicillin resistance gene; *aacC1*, gentamycin resistance gene.
(TIF)

Table S1 **Primers used in the present study.**
(DOCX)

## References

[1] Furlow B (2010). Tuberculosis: a review and update.. Radiol Technol.

[2] Jagielski T, Augustynowa-Kopec E, Zwolska Z (2010). Epidemiology of tuberculosis: a global, European and Polish perspective.. Wiad Lek.

[3] WHO (2010). Global Tuberculosis Control: World Health Organization.

[4] Chakroborty A Drug-resistant tuberculosis: an insurmountable epidemic?.

[5] Jassal M, Bishai WR (2009). Extensively drug-resistant tuberculosis.. Lancet Infect Dis.

[6] Harries AD, Dye C (2006). Tuberculosis.. Ann Trop Med Parasitol.

[7] Koul A, Arnoult E, Lounis N, Guillemont J, Andries K (2011). The challenge of new drug discovery for tuberculosis.. Nature.

[8] Chernyshev A, Fleischmann T, Kohen A (2007). Thymidyl biosynthesis enzymes as antibiotic targets.. Appl Microbiol Biotechnol.

[9] Vertessy BG, Toth J (2009). Keeping uracil out of DNA: physiological role, structure and catalytic mechanism of dUTPases.. Acc Chem Res.

[10] Varga B, Barabas O, Takacs E, Nagy N, Nagy P (2008). Active site of mycobacterial dUTPase: structural characteristics and a built-in sensor.. Biochem Biophys Res Commun.

[11] Takacs E, Nagy G, Leveles I, Harmat V, Lopata A (2010). Direct contacts between conserved motifs of different subunits provide major contribution to active site organization in human and mycobacterial dUTPases.. FEBS Lett.

[12] Chan S, Segelke B, Lekin T, Krupka H, Cho US (2004). Crystal structure of the Mycobacterium tuberculosis dUTPase: insights into the catalytic mechanism.. J Mol Biol.

[13] Ladner RD (2001). The role of dUTPase and uracil-DNA repair in cancer chemotherapy.. Curr Protein Pept Sci.

[14] Whittingham JL, Leal I, Nguyen C, Kasinathan G, Bell E (2005). dUTPase as a platform for antimalarial drug design: structural basis for the selectivity of a class of nucleoside inhibitors.. Structure.

[15] Wilson PM, Fazzone W, LaBonte MJ, Lenz HJ, Ladner RD (2009). Regulation of human dUTPase gene expression and p53-mediated transcriptional repression in response to oxaliplatin-induced DNA damage.. Nucleic Acids Res.

[16] Wilson PM, Fazzone W, LaBonte MJ, Deng J, Neamati N (2008). Novel opportunities for thymidylate metabolism as a therapeutic target.. Mol Cancer Ther.

[17] Nyman PO (2001). Introduction. dUTPases.. Curr Protein Pept Sci.

[18] Mustafi D, Bekesi A, Vertessy BG, Makinen MW (2003). Catalytic and structural role of the metal ion in dUTP pyrophosphatase.. Proc Natl Acad Sci U S A.

[19] Fiser A, Vertessy BG (2000). Altered subunit communication in subfamilies of trimeric dUTPases.. Biochem Biophys Res Commun.

[20] Kovari J, Barabas O, Takacs E, Bekesi A, Dubrovay Z (2004). Altered active site flexibility and a structural metal-binding site in eukaryotic dUTPase: kinetic characterization, folding, and crystallographic studies of the homotrimeric Drosophila enzyme.. J Biol Chem.

[21] Horvath A, Vertessy BG (2010). A one-step method for quantitative determination of uracil in DNA by real-time PCR.. Nucleic Acids Res.

[22] Lari SU, Chen CY, Vertessy BG, Morre J, Bennett SE (2006). Quantitative determination of uracil residues in Escherichia coli DNA: Contribution of ung, dug, and dut genes to uracil avoidance.. DNA Repair (Amst).

[23] Merenyi G, Kovari J, Toth J, Takacs E, Zagyva I (2011). Cellular response to efficient dUTPase RNAi silencing in stable HeLa cell lines perturbs expression levels of genes involved in thymidylate metabolism.. Nucleosides Nucleotides Nucleic Acids.

[24] Huffman JL, Li H, White RH, Tainer JA (2003). Structural basis for recognition and catalysis by the bifunctional dCTP deaminase and dUTPase from Methanococcus jannaschii.. J Mol Biol.

[25] Johansson E, Bjornberg O, Nyman PO, Larsen S (2003). Structure of the bifunctional dCTP deaminase-dUTPase from Methanocaldococcus jannaschii and its relation to other homotrimeric dUTPases.. J Biol Chem.

[26] Helt SS, Thymark M, Harris P, Aagaard C, Dietrich J (2008). Mechanism of dTTP inhibition of the bifunctional dCTP deaminase: dUTPase encoded by Mycobacterium tuberculosis.. J Mol Biol.

[27] Bjornberg O, Neuhard J, Nyman PO (2003). A bifunctional dCTP deaminase-dUTP nucleotidohydrolase from the hyperthermophilic archaeon Methanocaldococcus jannaschii.. J Biol Chem.

[28] el-Hajj HH, Zhang H, Weiss B (1988). Lethality of a dut (deoxyuridine triphosphatase) mutation in Escherichia coli.. J Bacteriol.

[29] Gadsden MH, McIntosh EM, Game JC, Wilson PJ, Haynes RH (1993). dUTP pyrophosphatase is an essential enzyme in Saccharomyces cerevisiae.. EMBO J.

[30] Guillet M, Van Der Kemp PA, Boiteux S (2006). dUTPase activity is critical to maintain genetic stability in Saccharomyces cerevisiae.. Nucleic Acids Res.

[31] Sassetti CM, Boyd DH, Rubin EJ (2003). Genes required for mycobacterial growth defined by high density mutagenesis.. Mol Microbiol.

[32] Griffin JE, Gawronski JD, Dejesus MA, Ioerger TR, Akerley BJ (2011). High-resolution phenotypic profiling defines genes essential for mycobacterial growth and cholesterol catabolism.. PLoS Pathog.

[33] Pecsi I, Leveles I, Harmat V, Vertessy BG, Toth J (2010). Aromatic stacking between nucleobase and enzyme promotes phosphate ester hydrolysis in dUTPase.. Nucleic Acids Res.

[34] Barabas O, Pongracz V, Kovari J, Wilmanns M, Vertessy BG (2004). Structural insights into the catalytic mechanism of phosphate ester hydrolysis by dUTPase.. J Biol Chem.

[35] Johansson E, Fano M, Bynck JH, Neuhard J, Larsen S (2005). Structures of dCTP deaminase from Escherichia coli with bound substrate and product: reaction mechanism and determinants of mono- and bifunctionality for a family of enzymes.. J Biol Chem.

[36] Parish T, Stoker NG (2000). Use of a flexible cassette method to generate a double unmarked Mycobacterium tuberculosis tlyA plcABC mutant by gene replacement.. Microbiology 146 ( Pt.

[37] Toth J, Varga B, Kovacs M, Malnasi-Csizmadia A, Vertessy BG (2007). Kinetic mechanism of human dUTPase, an essential nucleotide pyrophosphatase enzyme.. J Biol Chem.

[38] Dos Vultos T, Mestre O, Tonjum T, Gicquel B (2009). DNA repair in Mycobacterium tuberculosis revisited.. FEMS Microbiol Rev.

[39] Springer B, Sander P, Sedlacek L, Hardt WD, Mizrahi V (2004). Lack of mismatch correction facilitates genome evolution in mycobacteria.. Mol Microbiol.

[40] Boshoff HI, Reed MB, Barry CE, Mizrahi V (2003). DnaE2 polymerase contributes to in vivo survival and the emergence of drug resistance in Mycobacterium tuberculosis.. Cell.

[41] Varga B, Barabas O, Kovari J, Toth J, Hunyadi-Gulyas E (2007). Active site closure facilitates juxtaposition of reactant atoms for initiation of catalysis by human dUTPase.. FEBS Lett.

[42] Pecsi I, Szabo JE, Adams SD, Simon I, Sellers JR (2011). Nucleotide pyrophosphatase employs a P-loop-like motif to enhance catalytic power and NDP/NTP discrimination.. Proc Natl Acad Sci U S A.

[43] Raghunand TR, Bishai WR (2006). Mapping essential domains of Mycobacterium smegmatis WhmD: insights into WhiB structure and function.. J Bacteriol.

[44] Sinha KM, Glickman MS, Shuman S (2009). Mutational analysis of Mycobacterium UvrD1 identifies functional groups required for ATP hydrolysis, DNA unwinding, and chemomechanical coupling.. Biochemistry.

[45] Tormo-Mas MA, Mir I, Shrestha A, Tallent SM, Campoy S (2010). Moonlighting bacteriophage proteins derepress staphylococcal pathogenicity islands.. Nature.

[46] Sassetti CM, Rubin EJ (2003). Genetic requirements for mycobacterial survival during infection.. Proc Natl Acad Sci U S A.

[47] Sampathkumar P, Turley S, Sibley CH, Hol WG (2006). NADP+ expels both the co-factor and a substrate analog from the Mycobacterium tuberculosis ThyX active site: opportunities for anti-bacterial drug design.. J Mol Biol.

[48] Fivian-Hughes AS, Houghton J, Davis EO (2012). Mycobacterium tuberculosis thymidylate synthase gene thyX is essential and potentially bifunctional, while thyA deletion confers resistance to p-aminosalicylic acid.. Microbiology.

[49] Jarmula A (2010). Antifolate inhibitors of thymidylate synthase as anticancer drugs.. Mini Rev Med Chem.

[50] Cosma CL, Sherman DR, Ramakrishnan L (2003). The secret lives of the pathogenic mycobacteria.. Annu Rev Microbiol.

[51] Mustafa AS, Cockle PJ, Shaban F, Hewinson RG, Vordermeier HM (2002). Immunogenicity of Mycobacterium tuberculosis RD1 region gene products in infected cattle.. Clin Exp Immunol.

[52] Arnold K, Bordoli L, Kopp J, Schwede T (2006). The SWISS-MODEL workspace: a web-based environment for protein structure homology modelling.. Bioinformatics.

[53] Melo F, Feytmans E (1998). Assessing protein structures with a non-local atomic interaction energy.. J Mol Biol.

[54] Benkert P, Biasini M, Schwede T (2011). Toward the estimation of the absolute quality of individual protein structure models.. Bioinformatics.

[55] Laskowski R A MMW, Moss D, Thornton J M (1993). PROCHECK: a program to check the stereochemical quality of protein structures.. J Appl Cryst 26,.

[56] Snapper SB, Melton RE, Mustafa S, Kieser T, Jacobs WR (1990). Isolation and characterization of efficient plasmid transformation mutants of Mycobacterium smegmatis.. Mol Microbiol.

[57] Roberts G, Muttucumaru DG, Parish T (2003). Control of the acetamidase gene of Mycobacterium smegmatis by multiple regulators.. FEMS Microbiol Lett.

[58] Mahenthiralingam E, Marklund BI, Brooks LA, Smith DA, Bancroft GJ (1998). Site-directed mutagenesis of the 19-kilodalton lipoprotein antigen reveals No essential role for the protein in the growth and virulence of Mycobacterium intracellulare.. Infect Immun.

[59] Parish T, Stoker NG (1998). Electroporation of mycobacteria.. Methods Mol Biol.

[60] Goude R, Parish T (2008). Electroporation of mycobacteria..

[61] TJ Silhavy, Berman, L M, Enquist, W L (1984). Experiments with gene fusions; Laboratory..

