# Supplementary figures and images for: The dUTPase Enzyme Is Essential in Mycobacterium smegmatis
Source: PLoS One. 2012 May 24;7(5):e37461. doi: 10.1371/journal.pone.0037461 (PMC3360063; doi:10.1371/journal.pone.0037461)

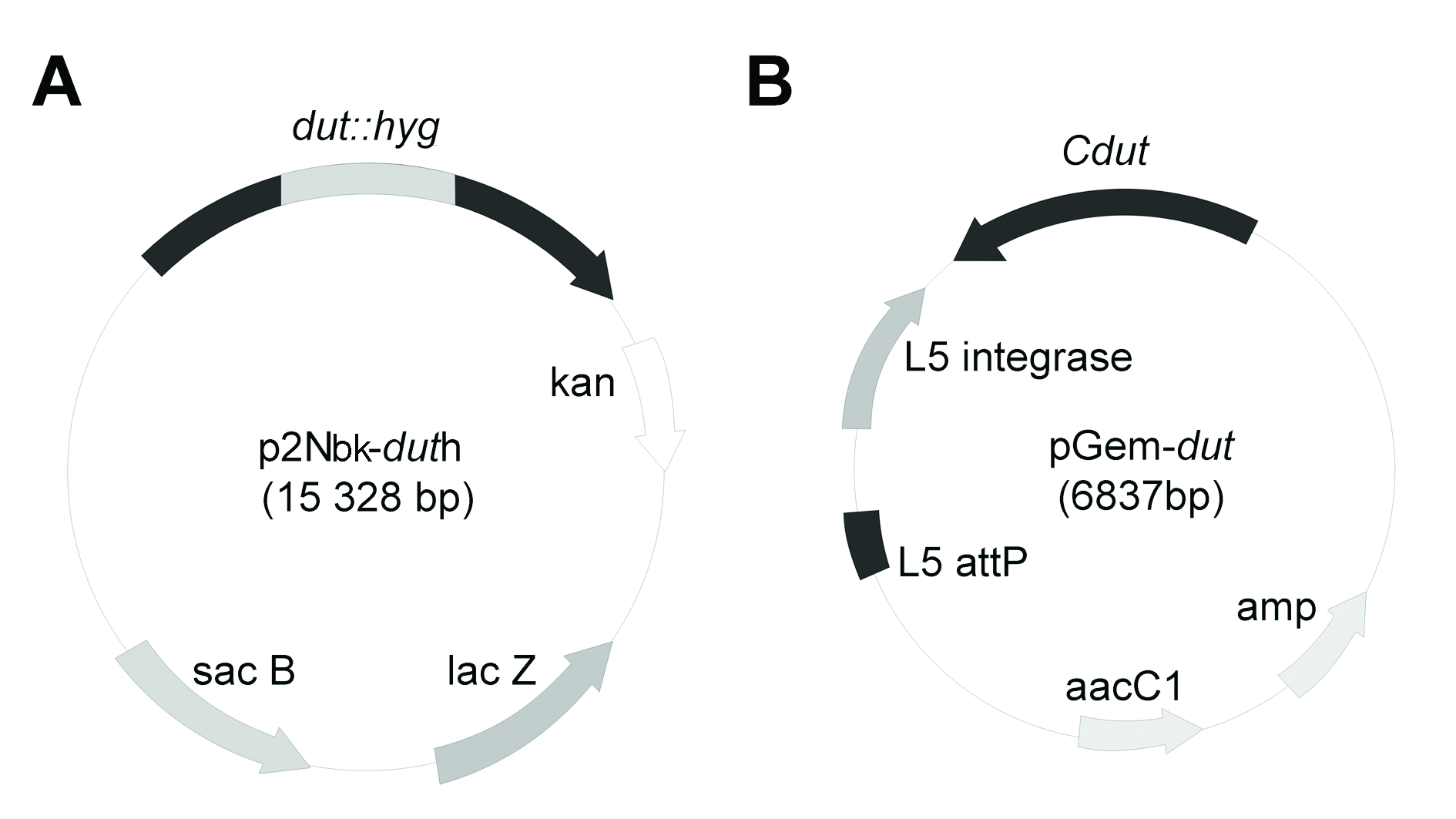

Supplement: Figure S1 — Key plasmids used in the generation of dut deletion mutant M. smegmatis . (A) p2Nbk-duth delivery vector used to generate mutant SCOs. The 2.1 kb HindIII M. smegmatis fragment indicated in Figure 3 was inserted into the p2NIL vector to construct the delivery vector. The dut allele was disrupted with a 1.8 kb fragment encoding hygromycin resistance, resulting in a non-functional dut gene. (B) The plasmid pGem-dut was used to complement the gene-disruption mutation. The wild-type dut allele together with its own promoter (337 bp upstream of the dut coding region) was cloned into an L5-based integrating vector to produce pGem-dut. Detailed cloning procedures are given in Materials and Methods. Cdut, WT dut gene with its own promoter; kan, kanamycin resistance gene; hyg, hygromycin resistance gene; lacZ, β – galactosidase; sacB, sucrose sensitivity gene; amp, ampicillin resistance gene; aacC1, gentamycin resistance gene. (TIF) [file pone.0037461.s001.tif]
