# Supplementary material for: The dUTPase Enzyme Is Essential in Mycobacterium smegmatis
Source: PLoS One. 2012 May 24;7(5):e37461. doi: 10.1371/journal.pone.0037461 (PMC3360063; doi:10.1371/journal.pone.0037461)
Supplement: Table S1 — Primers used in the present study. (DOCX) [file pone.0037461.s002.docx]

**Table S1.**

| **Cloning primer sequences (5'→3')** | **Function** |
| --- | --- |
| ctacgaagcttaccctgatcttggtctcggc | amplify dut gene with flanking regions for cloning p2NIL-*dut*h (HindIII site) |
| ctacgaagcttaccgagccgcgcgtgaccgg | amplify dut gene with flanking regions for cloning p2NIL-*dut*h (HindIII site) |
| cgtcaccggtgcagtcctccacgggcagctcgt | amplify *hyg* marker gene for disrupting *dut* (AgeI site) |
| cgtcaccggtctgaaggtggcatttccgcag | amplify *hyg* marker gene for disrupting *dut* (AgeI site) |
| cgccgatttcggcacctcgg | amplify *dut* gene with promoter region to cloning pGEM-*dut* |
| cgtcaagctttcacaaactcgcatgtccgcc | amplify *dut* gene with promoter region to cloning pGEM-*dut* (HindIII site) |
| cctcgttcgacgagacaacccgtggcg | quick-change mutagenesis primer for Δ-loop pGEM-*dut* |
| cgccacgggttgtctcgtcgaacgagg | quick-change mutagenesis primer for Δ-loop pGEM-*dut* |
| ctagctagctca*cttgtcatcgtcgtccttgtagtc*caaactcgcatgtc | flag-tag coding cloning primer for flag-tagged complement vectors (NheI site, *flag-tag*) |
| tactagctagctcgaccaagacgatcacc | cloning primer for flag-tagged complement vectors |
| gtgtcgaccactctggcg | amplify Mtb *dut* coding sequence for cloning pGEM-Mtb*dut* |
| ctagctagctca*cttgtcatcgtcgtccttgtagtc*caaactcgcatgtc | amplify Mtb *dut* coding sequence for cloning pGEM-Mtb*dut* (NheI site, *flag-tag*) |
| tactagctagctcgaccaagacgatcacc | amplify pGEM-*dut* vector without *dut* coding region (NheI site) |
| gacgtcacagattactctgagccg | amplify pGEM-*dut* vector without *dut* coding region |
| cgtcgttcgacgagacatcccgcggcgac | quick-change mutagenesis primer for Mtb Δ-loop *dut* |
| gtcgccgcgggatgtctcgtcgaacgacg | quick-change mutagenesis primer for Mtb Δ-loop *dut* |
| **Screening primer sequences (5'→3')** | **Function** |
| ggcggacctttccggagagg | amplify the region of the probe of the Southern blot |
| cgggggccagttcgacgttc | amplify the region of the probe of the Southern blot |
| acggcatacggaacccaggcc | SCO screening primer |
| gaaccaccagaaccatcggg | DCO screening primer |
| caccttcctgcacgacttcg | SCO/DCO screening primer |
| cgtctgcggatccaggttg | SCO/DCO screening primer |
| caacctggatccgcagacg | confirmation of integration of complement vector to the genome |
| ggctacgtctccgaactcacg | confirmation of integration of complement vector to the genome |
